# Poor Survival in Rheumatoid Arthritis Associated with Bronchiectasis: A Family-Based Cohort Study

**DOI:** 10.1371/journal.pone.0110066

**Published:** 2014-10-13

**Authors:** Xavier Puéchal, Emmanuelle Génin, Thierry Bienvenu, Claire Le Jeunne, Daniel J. Dusser

**Affiliations:** 1 National Referral Center for Rare Systemic Autoimmune Diseases, Department of Internal Medicine, Hôpital Cochin, Assistance Publique-Hôpitaux de Paris, Université Paris Descartes, Paris, France; 2 INSERM U1016, CNRS UMR 8104, Institut Cochin, Paris, France; 3 INSERM UMR-1078, Centre Hospitalier Universitaire, Brest, France; 4 Biochemistry and Molecular Biology Laboratory, Hôpital Cochin, Assistance Publique-Hôpitaux de Paris, Université Paris Descartes, Paris, France; 5 Department of Respiratory Diseases, Hôpital Cochin, Assistance Publique-Hôpitaux de Paris, Université Paris Descartes, Paris, France; Faculté de médecine de Nantes, France

## Abstract

**Background:**

Diffuse bronchiectasis (DB) may occur in rheumatoid arthritis (RA). *CFTR* (cystic fibrosis transmembrane conductance regulator) mutations predispose RA patients to DB, but the prognosis of RA-associated DB (RA-DB) is unclear.

**Methods:**

We report long-term mortality data from a nationwide family-based association study of patients with RA only, DB only or RA-DB. We assessed mortality as a function of clinical characteristics and CF/*CFTR*-RD (*CFTR*-related disorders) mutations in 137 subjects from 24 kindreds. Potential risk factors were investigated by Cox proportional-hazard analysis with shared Gaussian random effects to account for within-family correlations.

**Results:**

During a median follow-up of 11 years after inclusion, 18 patients died, mostly from cardiorespiratory causes. Survival was significantly lower for RA-DB patients than for unaffected relatives and for patients with RA or DB only. RA patients with DB had also a poorer prognosis in terms of survival after RA diagnosis (HR, 8.6; 95% CI, 1.5–48.2; *P* = 0.014) and from birth (HR, 9.6; 95% CI, 1.1–81.7; *P* = 0.039). Early onset of DB (HR, 15.4; 95% CI, 2.1–113.2; *P* = 0.007) and CF/*CFTR*-RD mutation (HR, 7.2; 95% CI, 1.4–37.1; *P* = 0.018) were associated with poorer survival in patients with RA-DB. Thus, CF/*CFTR*-RD mutations in RA patients with early-onset DB defined a subgroup of high-risk patients with higher mortality rates (log-rank test *P* = 1.28×10^−5^).

**Conclusion:**

DB is associated with poorer survival in patients with RA. Early-onset DB and *CFTR* mutations are two markers that identify RA patients at a high risk of death, for whom future therapeutic interventions should be designed and evaluated.

## Introduction

Rheumatoid arthritis (RA) is a systemic autoimmune disease, of unknown origin, that affects 0.5–1.0% of the adult population worldwide [1]. It primarily involves the joints, but extra-articular manifestations, such as lung involvement, have been observed and may increase the burden of this frequently disabling disease. Most studies in RA patients have reported higher mortality rates than for the general population and have shown that extra-articular manifestations contribute to premature death [2], [3].

There is a well-established association between RA and diffuse bronchiectasis (DB). In prospective blind studies based on high-resolution computed tomography (HRCT) in patients with RA, the prevalence of diffuse bronchiectasis has been reported to range from 5.6% to 30% [4]–[9]. Conversely, RA is found in 2.7% to 5.2% of patients with DB referred to respiratory medicine departments for investigation [10], [11]. In cases of symptomatic DB in RA patients, respiratory symptoms precede the onset of RA in more than 90% of cases, with a mean interval of more than 30 years between the onset of the two diseases (early-onset, mostly childhood DB) [10], [12]–[16]. In less than 10% of patients, respiratory symptoms of DB occur concomitantly with the onset of arthritis (late-onset and adulthood DB) [17]. The prognosis of these two subtypes of DB in RA patients remains unclear. Recent data have suggested that the lung may be an early site of autoimmune-related injury in RA patients and a potential site involved in RA-related autoimmunity [18], [19]. Further analyses of the link between RA and DB are, therefore, required.

Cystic fibrosis transmembrane conductance regulator (*CFTR*) gene mutations have been identified as the cause of cystic fibrosis (CF) [20], a disease associated with DB and impaired survival due to respiratory insufficiency in most cases [21]. Beside CF, some DB may be classified as *CFTR*-related diseases (*CFTR*-RD), defined as a clinical entity associated with CFTR dysfunction not fulfilling the diagnostic criteria for CF [22].

We previously reported a higher prevalence of CF/*CFTR*-RD mutations in patients suffering from RA-associated DB (RA-DB) than in the general population [16]. We found a strong association and linkage between mutations in the CF/*CFTR*-RD gene and RA-DB phenotypes in a French nationwide family-based association study [23]. CF/*CFTR*-RD mutations were five times more frequent in RA-DB patients than in patients with RA only (no DB) and cosegregated with RA-DB in the families. Furthermore, RA-DB patients carrying at least one abnormal *CFTR* allele but not fulfilling the diagnostic criteria for CF more frequently had chronic sinusitis, a trend toward more severe pulmonary involvement, and CFTR protein dysfunction, as demonstrated by the lower nasal potential difference of these patients than of patients with both RA and DB but without CF/*CFTR*-RD mutations [16]. These data suggest that CF/*CFTR*-RD mutations may predispose patients with RA to DB, but the vital prognosis of the RA-DB association has not been investigated in detail, and the influence of the clinical onset of DB and the presence of CF/*CFTR*-RD mutations on survival has not been studied.

We therefore investigated long-term mortality and variables predictive of the risk of death in a well characterized, prospectively followed family-based cohort of patients with a diagnosis of RA only, DB only, or RA-DB.

## Methods

The study protocol was reviewed and approved by the institutional review board for clinical research of *Assistance Publique-Hôpitaux de Paris* and the ethics committee of the Medical Faculty of Paris Descartes University. Written informed consent was obtained from all participants.

### Study population

The study was performed in a prospectively followed family-based cohort recruited between September 1999 and February 2002, which has been described elsewhere [23]. Briefly, for inclusion in the study, each family had to include at least one proband with both RA and symptomatic DB and one affected first-degree relative with RA and/or DB. Once a family was deemed eligible for inclusion, all affected subjects and unaffected relatives were interviewed and blood samples were taken for *CFTR* genotyping.

### Interview and clinical assessment at inclusion

At inclusion, pedigree analysis was carried out and each family member underwent face-to-face semi-structured interviews and clinical assessment. Given the known association of RA and DB and the possibility of asymptomatic or paucisymptomatic DB, all RA patients underwent HRCT of the lungs. An HRCT scan of the lungs was also conducted for other participants presenting respiratory symptoms. All cases of DB were confirmed by two independent observers finding evidence of diffuse (more than one lobe) bronchiectasis on HRCT scan, without knowledge of the *CFTR* mutational status of the individual concerned. In patients with both RA and DB, DB was defined as early-onset DB if respiratory symptoms preceded RA by more than 10 years (mostly childhood DB) and as late-onset DB for patients whose respiratory symptoms occurred at the same time or less than 10 years before RA onset (adulthood DB). Participants were classified into four categories: RA-DB, RA without DB (RA only), DB only and unaffected.

### *CFTR* genotyping

We checked the entire coding region and exon/intron junctions of *CFTR* for CF/*CFTR*-RD mutations, which were subsequently confirmed by direct DNA sequencing, as previously described [23]. Nucleotide substitutions were classified according to their known or predicted functional consequences [23] and groups of mutations were considered collectively in the analyses.

### Survival

The primary outcome measure was survival between November 2011 and December 2012. Data were censored on December 31^st^, 2012. Vital status was recorded in this family-based cohort, with a new questionnaire that was sent to each participant and a follow-up interview carried out for all families. Information about the cause of death was collected retrospectively from a family questionnaire and medical reports. Survival from inclusion was determined for all participants and survival from birth and from RA diagnosis was also analyzed.

### Statistical analysis

The characteristics of the patients (sex ratio, age at inclusion, age at diagnosis, median follow-up) were compared between the groups in chi-squared tests, Fisher's exact tests or Kruskal-Wallis tests, as appropriate.

Survival analysis was performed with the R package “survival” [24]. Kaplan-Meier survival curves were plotted with the “plot.survfit” function and the log-rank test, implemented in the “survdiff” function, was used to compare survival between groups.

We adjusted for the effect of covariates and estimated hazard ratios (HR) while accounting for family correlations, by fitting Cox models with Gaussian random effects models to the data with the R package “coxme” [25]. Each individual was assumed to have a random genetic risk, but risks were correlated between individuals according to the strength of their pedigree relationship, as assessed by their kinship coefficient. We report HR and 95% confidence intervals (95% CI) and the *P*-value of the *z* test.

We compared survival from inclusion, adjusted for age at inclusion, between the individuals included in the study, as a function of their disease status. We then focused on RA patients and compared their survival from birth and from RA diagnosis as a function of whether or not they had DB (comparison of RA-DB with RA only). Finally, we focused on patients with both RA and DB and investigated the factors potentially influencing their survival from birth or from RA diagnosis.

All analyses were conducted with R version 2.15.1 [26].

## Results

### Patients' characteristics

The family-based cohort comprised 137 subjects from 24 kindreds, including 30 patients with RA-DB, 25 patients with RA only and eight patients with DB only; the remaining participants constituted the unaffected relatives.

The main demographic features of the participants enrolled in the family-based cohort are shown in Table 1. The median follow-up time after inclusion was 10, 11, 11, and 12 years for patients with RA-DB, RA only, DB only, and unaffected relatives, respectively.

**Table 1:** Characteristics and outcome of the 137 participants in the family-based cohort study.

Characteristics	RA-DB	RA only	DB only	Unaffected relatives	*P* value
	(*N* = 30)	(*N* = 25)	(*N* = 8)	(*N* = 74)	
Sex ratio (F/M)	25/5	17/8	5/3	37/37	0.011^a^
Median (mean) age at RA onset − yr	45 (45.1)	39 (42.0)	-	-	0.35^b^
Median (mean) age at inclusion − yr	64.0 (60.1)	51.0 (51.1)	45.5 (50.1)	52.0 (54.7)	0.08^b^
Symptoms at inclusion					
Dyspnea − no. (%)	24 (80%)		7 (86%)		1^a^
Cough and sputum − no. (%)	24 (80%)		7 (86%)		1^a^
>1 respiratory infection/year − no. (%)	19 (63%)		6 (71%)		0.69^a^
Sinus involvement − no. (%)	14 (47%)		5 (63)%		0.69^a^
Hemoptysis − no. (%)	7 (23%)		3 (38%)		0.41^a^
Subjects with at least one *CFTR* mutation − no. (%)	18 (60%)	5 (20%)	6 (75%)	16 (22%)	5.5×10^−5^ a
Age when last seen − yr					0.15^b^
Median	70.5	62	56	65.5	
Mean+SD	69.57+9.74	62.80+12.70	61.00+17.74	67.07+14.45	
Median (mean) follow-up time − yr	10 (9.5)	11 (11.7)	11 (10.9)	12 (12.4)	0.06^b^
Deaths − no. (%)	14 (46.7%)	2 (8.0%)	2 (25.0%)	11 (14.9%)	0.0015^a^
Cause of death – no.					
cardiorespiratory	8	1		1	
others	4*	1†	1‡	5¥	
unknown	2		1	5	

RA: rheumatoid arthritis; DB: diffuse bronchiectasis. *CFTR*: cystic fibrosis transmembrane conductance regulator gene.

Plus-minus values are means+SD.

a *P*-values of Fisher exact tests.

b *P*-values of Kruskal-Wallis tests.

* Causes of death included CVA, Hodgkin lymphoma, cancer, and refractory RA, in one patient each.

† Cause of death was CVA.

‡ Cause of death was cancer.

¥ Causes of death included cancer in 4 patients and degenerative neurological disease in one patient.

### Survival in different groups of individuals

At the time of analysis, vital status was known for all subjects. Eighteen patients and 11 unaffected relatives from the study cohort had died (Table 1). In patients with RA-DB, the reported cause of death was identified in 12 of the 14 patients who died, and cardiorespiratory disease was the principal cause of death reported for eight of these patients.

**Figure 1:**
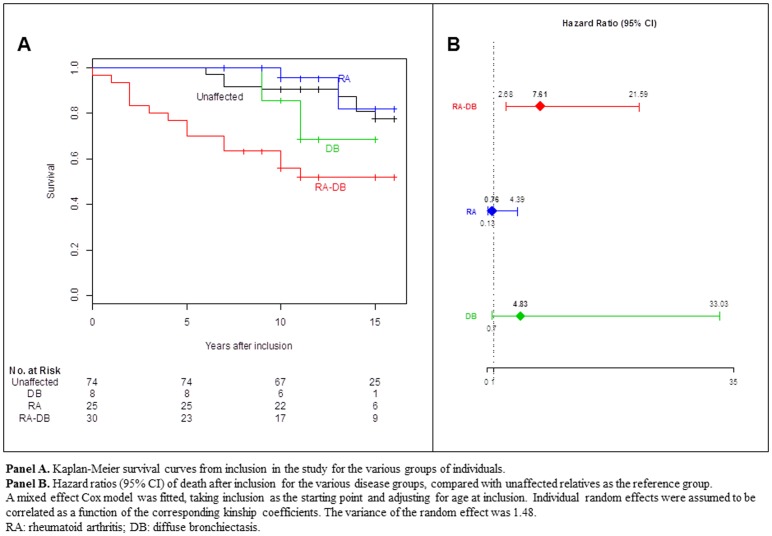
Kaplan-Meier probability of survival from inclusion of the various participants in the study cohort, according to their phenotype. **Panel A.** Kaplan-Meier survival curves from inclusion in the study for the various groups of individuals. **Panel B.** Hazard ratios (95% CI) of death after inclusion for the various disease groups, compared with unaffected relatives as the reference group. A mixed effect Cox model was fitted, taking inclusion as the starting point and adjusting for age at inclusion. Individual random effects were assumed to be correlated as a function of the corresponding kinship coefficients. The variance of the random effect was 1.48. RA: rheumatoid arthritis; DB: diffuse bronchiectasis.

There were more deaths after inclusion among the patients with RA-DB than among the other groups (RA only, DB only, unaffected relatives) (Table 1), even after adjustment for age at inclusion (Fig. 1A). RA-DB patients tended to be older at inclusion than patients with RA only or DB only (Table 1). We therefore also compared survival from birth between the different groups (Fig. S1A). For patients with RA or DB only, survival after inclusion or since birth was not significantly different from the survival of unaffected relatives (log-rank test *P* = 0.71 and *P* = 0.21, respectively). By contrast, survival was a mean of 8.6 years shorter for RA-DB patients than for unaffected relatives (log-rank test *P* = 6.2×10^−5^) (Fig. 1A and Fig. S1A). In mixed effect Cox models, with unaffected relatives as the reference group, the HR for death was significant only for patients with RA-DB, whether we took inclusion in the study (HR, 7.61; 95% CI, 2.68–21.59; *z*-test *P* = 0.00014) (Fig. 1B) or birth as the starting points (Fig. S1B).

### Survival in rheumatoid arthritis patients with and without bronchiectasis

**Figure 2:**
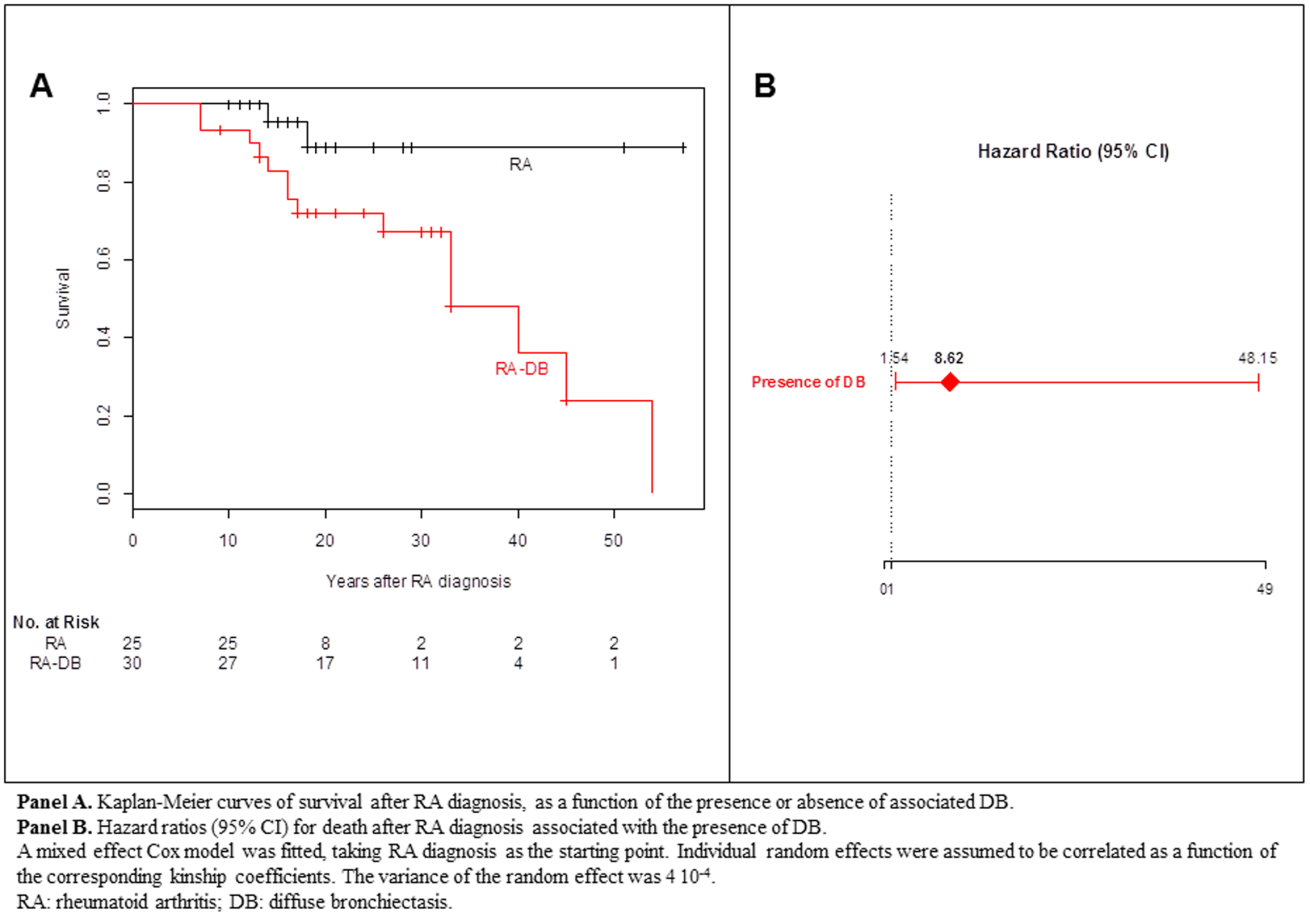
Kaplan-Meier probability of survival after RA diagnosis in patients, as a function of the presence or absence of associated DB. **Panel A**. Kaplan-Meier curves of survival after RA diagnosis, as a function of the presence or absence of associated DB. **Panel B**. Hazard ratios (95% CI) for death after RA diagnosis associated with the presence of DB. A mixed effect Cox model was fitted, taking RA diagnosis as the starting point. Individual random effects were assumed to be correlated as a function of the corresponding kinship coefficients. The variance of the random effect was 4 10^−4^. RA: rheumatoid arthritis; DB: diffuse bronchiectasis.

The survival of RA-DB patients after RA diagnosis was significantly lower than that of patients with RA only, even after adjusting for age of diagnosis (Fig. 2). Kaplan-Meier estimates of the probability of survival 15 years after RA diagnosis were 82.8% (95% CI, 70.2% to 97.8%) for RA-DB patients and 95.2% (95% CI, 86.6% to 100%) for patients with RA only (*P* = 0.022). In the mixed effect Cox model adjusted for age at RA diagnosis, the HR for death associated with the presence of DB was 8.6 (95% CI, 1.5 to 48.2; *z*-test P = 0.014) (Fig. 2B). We obtained similar results if we considered survival from birth, with a HR for death associated with the presence of DB of 9.6 (95% CI, 1.1 to 81.7; *z*-test *P* = 0.039).

### Factors influencing survival in patients with both rheumatoid arthritis and bronchiectasis

We found that 18 of the 30 patients with RA-DB (60%) had early-onset DB, whereas the other 12 patients with RA-DB (40%) had late-onset DB. Individuals with early-onset DB tended to be diagnosed with RA at younger ages than those with late-onset DB. We therefore studied survival from birth in RA-DB patients.

In univariate analyses of survival from birth, early-onset DB, CF/*CFTR*-RD mutations and being male were associated with poorer survival (Table 2).

**Table 2:** Analysis of survival from birth of 30 patients with both RA and DB.

	Univariate analysis	Multivariate analysis	
	*P* value	Hazard ratio [95% CI]	*P* value
**Early-onset DB**	0.009	15.4 [2.1; 113.2]	0.0072
***CFTR*** ** mutations**	0.073	7.2 [1.4; 37.1]	0.018
**Male versus female**	0.06	1.1 [0.2; 5.6]	0.92

RA: rheumatoid arthritis; DB: diffuse bronchiectasis.

Early-onset DB: respiratory symptoms preceding RA by >10 years (mostly childhood DB).

*CFTR*: cystic fibrosis transmembrane conductance regulator gene.

A mixed effect Cox model was used, with shared random effects, depending on the kinship coefficient of individuals.

The variance of the random effect was 4×10^−4^.

**Figure 3:**
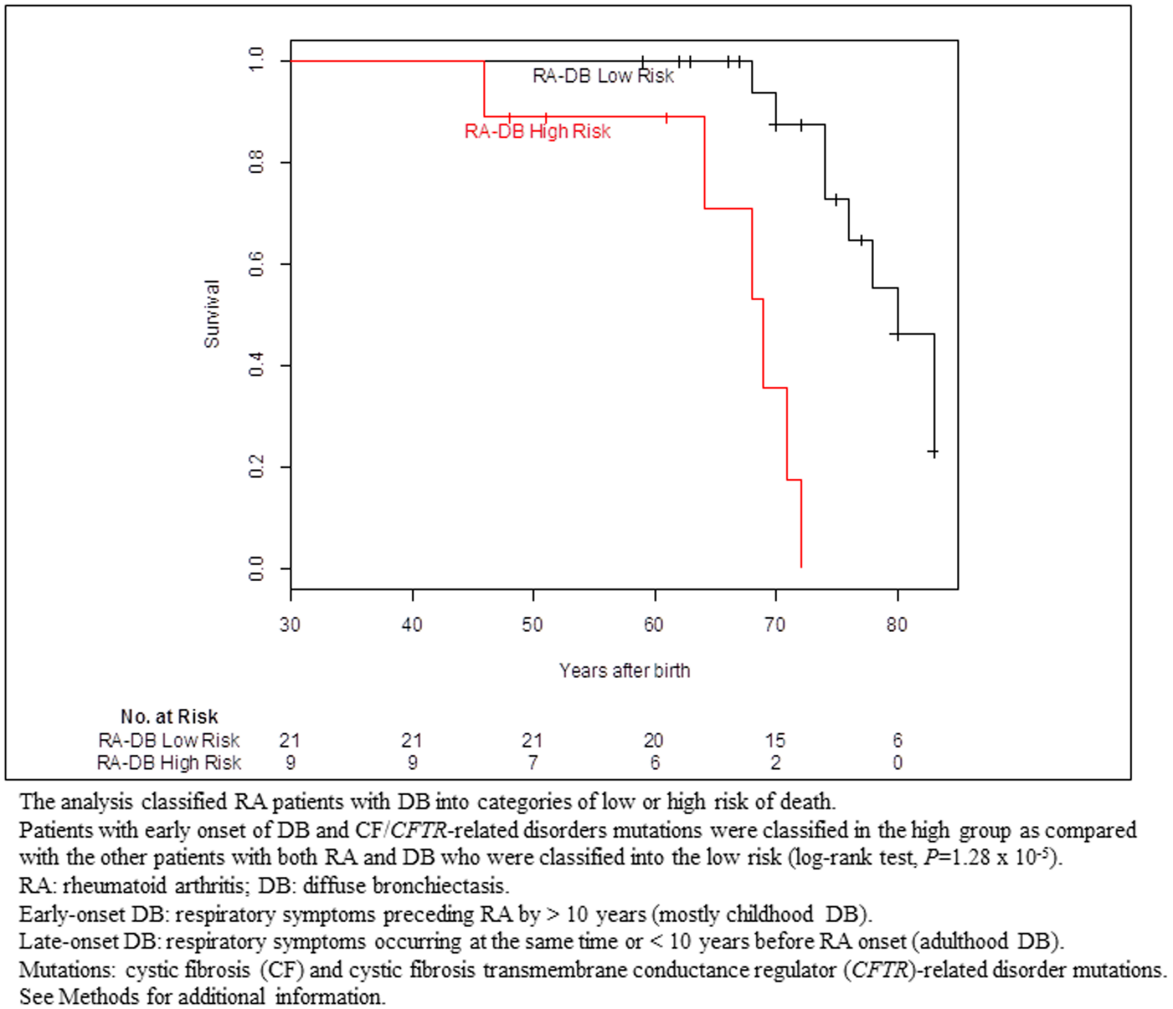
Kaplan-Meier probability of survival from birth of patients with both RA and DB according to risk of death categories. The analysis classified RA patients with DB into categories of low or high risk of death. Patients with early onset of DB and CF/*CFTR*-related disorders mutations were classified in the high group as compared with the other patients with both RA and DB who were classified into the low risk. RA: rheumatoid arthritis; DB: diffuse bronchiectasis. Early-onset DB: respiratory symptoms preceding RA by >10 years (mostly childhood DB). Late-onset DB: respiratory symptoms occurring at the same time or <10 years before RA onset (adulthood DB). Mutations: cystic fibrosis (CF) and cystic fibrosis transmembrane conductance regulator (*CFTR*)-related disorder mutations. See Patients and Methods for additional information.

Multivariate analysis of survival from birth showed that, in patients with RA-DB, an early onset of DB (HR, 15.4; 95% CI, 2.1 to 113.2; *P* = 0.0072) and CF/*CFTR*-RD mutations (HR, 7.2; 95% CI, 1.4 to 37.1; *P* = 0.018) were the two major determinants associated with poorer survival (Table 2). Eight of the 14 RA-DB patients who died (57.1%) carried CF/*CFTR*-RD mutations and 10 (71.4%) had early-onset DB. The corresponding figures were six of 16 (37.5%) and 8 of 16 (50%) for the surviving RA-DB patients. The analysis classified RA patients with DB into categories of low or high risk of death. CF/*CFTR*-RD mutations in RA patients with early-onset DB defined a subgroup of high-risk patients with higher mortality rates (log-rank test *P* = 1.28×10^−5^). Only RA patients with both an early onset of DB and CF/*CFTR*-RD mutations were found to have significantly poorer survival and these patients tended to develop RA at a younger age than the other RA-DB patients. Survival from birth did not differ significantly between patients with late-onset DB and no mutation, patients with late-onset DB and CF/*CFTR*-RD mutations and patients with early-onset DB and no mutation (log-rank test  = 2.8; 2 df; *P* = 0.251). Survival in RA-DB patients is shown according to risk of death categories in Figure 3.

## Discussion

In this prospective cohort study, DB was associated with poorer survival in RA patients. This analysis further identified early-onset DB and CF/*CFTR*-RD mutation as factors associated with a poorer survival in RA-DB patients.

This study provides the first evidence of long-term poor survival in patients with DB-associated RA, who were found to have an eight times higher probability of death after RA diagnosis than family members with RA only. Cardiorespiratory causes were the most frequently reported causes of death in this population. These results are similar to those of a previous controlled study, in which the five-year probability of mortality in patients with RA-DB was found to be five times that of a matched control group of patients with RA alone and 2.4 times that of a group of patients with DB alone [14]. Most of the deaths of patients with RA-DB (60%) in this previous study were attributed to respiratory infection, whereas the severity of RA, as assessed by HAQ, grip strength, nodules and steroid treatment, was not significantly associated with poorer survival [14].

In patients with RA, childhood-onset DB is much more prevalent than late-onset DB [10], [12]-[16]. Most (60%) of the 30 patients from our cohort with both RA and DB had early-onset DB, beginning more than 10 years before the onset of RA. These data are consistent with the hypothesis that the lung may be an early target of systemic RA autoimmunity or a site at which RA-related autoimmunity is initiated, with an initial autoimmune response in the lung [18], [19]. We found that RA-DB patients with early-onset DB had a poorer prognosis than those with late-onset DB, and early-onset DB was identified as a major determinant of survival in RA patients in multivariate analysis. The role of CF/*CFTR*-RD mutation in the association between RA and DB has been described before [23] and such mutations were found in 50% of the RA-DB patients with early-onset DB and 42% of those with late-onset DB. Not only do CF/*CFTR*-RD mutations predispose RA patients to DB, they are also associated with poorer survival. The prognostic implications of *CFTR* gene mutations have been considered to date only for in patients with CF. Our results demonstrate that RA patients with both early-onset DB and CF/*CFTR*-RD mutations should be considered at high risk of death and should receive more aggressive treatment, including smoking cessation and physiotherapy. Most of our patients were enrolled before the widespread use of biological agents likely to modify mortality [27], but frequent pulmonary superinfections may constitute a contraindication for such treatments in these patients [15]. These high-risk patients should be eligible in priority in protocols evaluating new treatment interventions such as long-term macrolide therapy which may be beneficial in patients with non-CF bronchiectasis [28]–[33] and significantly improve the signs and symptoms of RA [34], [35]. It remains unclear which patients should receive antibiotic treatment [36], but we suggest that RA patients with early-onset DB carrying CF/*CFTR*-RD mutations should be among those treated and evaluated for long-term azithromycin therapy.

Our study has several strengths. It is a family-based study, which made it possible to gather long-term data for all the individuals included. Such a family-based design limits research bias, by providing appropriate controls and follow-up. Furthermore, phenotypic characterization was carried out and mutational *CFTR* status was determined for all individuals. We systematically checked for DB on HRCT scans for all RA patients and for all individuals with respiratory symptoms. These scans were assessed by two investigators blind to the results of genetic tests.

Several limitations of this study merit further comment. The predictors of mortality in RA include severe extra-articular manifestations [2], [37]–[41]. One of the limitations of this nationwide study is the lack of information regarding other risk factors for mortality in RA patients. These confounding factors may influence the outcome, but the strongest predictor of mortality has been shown to be extra-articular manifestations of the disease [2], [40] and the leading cause of death of our RA-DB patients was cardiorespiratory complications. Sample size is another potential limitation of this study. The limited number of patients in each subgroup restricts the power of this study to detect differences. However, this series of patients with RA-DB is one of the largest ever studied, and differences in age at onset of DB and genetic background appear to have a significant, independent, and strong effect on mortality. In addition, our patients were selected from families with a history of RA or DB. It therefore remains unclear to what extent these results can be extrapolated to patients with no such family history. Another limitation of this study is the possible overestimation of the survival of patients with RA and late-onset DB with respect to that of patients with RA and early-onset DB. Indeed, for recruitment in the study, a family had to include at least one patient with RA and DB. Thus, if this patient had late-onset DB, there is the possibility of a survival advantage because he or she must have lived long enough after the onset of RA to develop DB meeting the late-onset criterion. However, this should not have biased the results concerning the deleterious effects of CF/*CFTR*-RD mutations on survival in patients with early-onset DB, in whom the presence of a mutation was found to lead to a mean decrease in survival of 7.1 years on average (log rank test  = 6.3; 1 df; *P* = 0.012).

## Conclusions

In this prospective family cohort, DB in RA patients was associated with lower overall survival. CF/*CFTR*-RD mutations predispose RA patients with early-onset DB to more severe disease, associated with a shorter lifespan. The identification of early-onset DB and the genotyping of such patients may make it possible to define high-risk patients for whom new treatments should be developed and evaluated specifically.

## Supporting Information

Figure S1 **Kaplan-Meier probability of survival from birth of the various participants in the study cohort as a function of their phenotype. Panel A.** Kaplan-Meier survival curves from birth for the various groups of individuals. **Panel B.** Hazard ratios (95% CI) for death since birth for the various disease groups, compared with unaffected relatives as the reference group. A mixed effect Cox model was fitted, taking birth as the starting point and age as the scale. Individual random effects were assumed to be correlated as a function of the corresponding kinship coefficient. The variance of the random effect was 1.35. RA: rheumatoid arthritis; DB: diffuse bronchiectasis.
(TIF)

## References

[1] Firestein GS (2003) Evolving concepts of rheumatoid arthritis. Nature 423: 356–361. 12748655 10.1038/nature01661

[2] Turesson C, O'Fallon WM, Crowson CS, Gabriel SE, Matteson EL (2002) Occurrence of extraarticular disease manifestations is associated with excess mortality in a community based cohort of patients with rheumatoid arthritis. J Rheumatol 29: 62–67. 11824973

[3] Aviña-Zubieta JA, Choi HK, Sadatsafavi M, Etminan M, Esdaile JM, et al (2008) Risk of cardiovascular mortality in patients with rheumatoid arthritis: a meta-analysis of observational studies. Arthritis Rheum 59: 1690–1697. 19035419 10.1002/art.24092

[4] Fewins HE, McGowan I, Whitehouse GH, Williams J, Mallya R (1991) High definition computed tomography in rheumatoid arthritis associated pulmonary disease. Br J Rheumatol 30: 214–216. 2049584 10.1093/rheumatology/30.3.214

[5] McDonagh J, Greaves M, Wright AR, Heycock C, Owen JP, et al (1994) High resolution computed tomography of the lungs in patients with rheumatoid arthritis and interstitial lung disease. Br J Rheumatol 33: 118–122. 8162474 10.1093/rheumatology/33.2.118

[6] Hassan WU, Keaney NP, Holland CD, Kelly CA (1995) High resolution computed tomography of the lung in lifelong non-smoking patients with rheumatoid arthritis. Ann Rheum Dis 54: 308–310. 7763110 10.1136/ard.54.4.308 PMC1005579

[7] Vergnenègre A, Pugnere N, Antonini MT, Arnaud M, Melloni B, et al (1997) Airway obstruction and rheumatoid arthritis. Eur Respir J 10: 1072–1078. 9163649 10.1183/09031936.97.10051072

[8] Perez T, Remy-Jardin M, Cortet B (1998) Airways involvement in rheumatoid arthritis: clinical, functional, and HRCT findings. Am J Respir Crit Care Med 157: 1658–1665. 9603152 10.1164/ajrccm.157.5.9710018

[9] Zrour SH, Touzi M, Bejia I, Golli M, Rouatbi N, et al (2005) Correlations between high-resolution computed tomography of the chest and clinical function in patients with rheumatoid arthritis: prospective study in 75 patients. Joint Bone Spine 72: 41–47. 15681247 10.1016/j.jbspin.2004.02.001

[10] Solanki T, Neville E (1992) Bronchiectasis and rheumatoid disease: is there an association? Br J Rheumatol 31: 691–693. 1393376 10.1093/rheumatology/31.10.691

[11] Pasteur MC, Helliwell SM, Houghton SJ, Webb SC, Foweraker JE, et al (2000) An investigation into causative factors in patients with bronchiectasis. Am J Respir Crit Care Med 162: 1277–1284. 11029331 10.1164/ajrccm.162.4.9906120

[12] Mathieu JP, Stack BH, Dick WC, Buchanan WW (1978) Pulmonary infection and rheumatoid arthritis. Br J Dis Chest 72: 57–61. 623705 10.1016/0007-0971(78)90007-4

[13] McMahon MJ, Swinson DR, Shettar S, Wolstenholme R, Chattopadhyay C, et al (1993) Bronchiectasis and rheumatoid arthritis: a clinical study. Ann Rheum Dis 52: 776–779. 8250608 10.1136/ard.52.11.776 PMC1005187

[14] Swinson DR, Symmons D, Suresh U, Jones M, Booth J (1997) Decreased survival in patients with co-existent rheumatoid arthritis and bronchiectasis. Br J Rheumatol 36: 689–691. 9236680 10.1093/rheumatology/36.6.689

[15] Puéchal X (1998) Bronchiectasis in rheumatoid arthritis. Rev Rhum Engl Ed 65: 447–450. 9785389

[16] Puéchal X, Fajac I, Bienvenu T, Desmazes-Dufeu N, Hubert D, et al (1999) Increased frequency of cystic fibrosis deltaF508 mutation in bronchiectasis associated with rheumatoid arthritis. Eur Respir J 13: 1281–1287. 10445602 10.1183/09031936.99.13612889

[17] Shadick NA, Fanta CH, Weinblatt ME, O'Donnell W, Coblyn JS (1994) Bronchiectasis. A late feature of severe rheumatoid arthritis. Medicine (Baltimore) 73: 161–170. 8190039

[18] Demoruelle MK, Weisman MH, Simonian PL, Lynch DA, Sachs PB, et al (2012) Airways abnormalities and rheumatoid arthritis-related autoantibodies in subjects without arthritis: early injury or initiating site of autoimmunity? Arthritis Rheum 64: 1756–1761. 22183986 10.1002/art.34344 PMC3319006

[19] Reynisdottir G, Karimi R, Joshua V, Olsen H, Hensvold AH, et al (2014) Structural changes and antibody enrichment in the lungs are early features of anti-citrullinated protein antibody-positive rheumatoid arthritis. Arthritis Rheumatol 66: 31–39. 24449573 10.1002/art.38201

[20] Knowles MR, Durie PR (2002) What is cystic fibrosis? N Engl J Med 347: 439–442. 12167688 10.1056/NEJMe020070

[21] McKone EF, Emerson SS, Edwards KL, Aitken ML (2003) Effect of genotype on phenotype and mortality in cystic fibrosis: a retrospective cohort study. Lancet 361: 1671–1676. 12767731 10.1016/S0140-6736(03)13368-5

[22] Bombieri C, Claustres M, De Boeck K, Derichs N, Dodge J, et al (2011) Recommendations for the classification of diseases as CFTR-related disorders. J Cyst Fibros 10 Suppl 2 S86–S102. 21658649 10.1016/S1569-1993(11)60014-3

[23] Puéchal X, Bienvenu T, Génin E, Berthelot JM, Sibilia J, et al (2011) Mutations of the cystic fibrosis gene in patients with bronchiectasis associated with rheumatoid arthritis. Ann Rheum Dis 70: 653–659. 21131649 10.1136/ard.2010.142760

[24] Therneau T (2013) A package for survival analysis in S. R package version 2.37–4. Available: http://CRAN.R-project.org/package=survival. Accessed 2014 Sep 19.

[25] Therneau T (2012) Coxme: Mixed Effects Cox Models. R package version 2.2–3. Available: http://CRAN.R-project.org/package=coxme. Accessed 2014 Sep 19.

[26] R Core Team (2012) R: A language and environment for statistical computing. R Foundation for Statistical Computing, Vienna, Austria. ISBN 3-900051-07-0. Available: http://www.R-project.org/. Accessed 2014 Sep 19.

[27] Jacobsson LT, Turesson C, Nilsson JA, Petersson IF, Lindqvist E, et al (2007) Treatment with TNF blockers and mortality risk in patients with rheumatoid arthritis. Ann Rheum Dis 66: 670–675. 17158824 10.1136/ard.2006.062497 PMC1954627

[28] Koh YY, Lee MH, Sun YH, Sung KW, Chae JH (1997) Effect of roxithromycin on airway responsiveness in children with bronchiectasis: a double-blind, placebo-controlled study. Eur Respir J 10: 994–999. 9163637 10.1183/09031936.97.10050994

[29] Tsang KW, Ho PI, Chan KN, Ip MS, Lam WK, et al (1999) A pilot study of low-dose erythromycin in bronchiectasis. Eur Respir J 13: 361–364. 10065682 10.1183/09031936.99.13236199

[30] Wong C, Jayaram L, Karalus N, Eaton T, Tong C, et al (2012) Azithromycin for prevention of exacerbations in non-cystic fibrosis bronchiectasis (EMBRACE): a randomised, double-blind, placebo-controlled trial. Lancet 380: 660–667. 22901887 10.1016/S0140-6736(12)60953-2

[31] Altenburg J, de Graaff CS, Stienstra Y, Sloos JH, van Haren EH, et al (2013) Effect of azithromycin maintenance treatment on infectious exacerbations among patients with non-cystic fibrosis bronchiectasis: the BAT randomized controlled trial. JAMA 309: 1251–1259. 23532241 10.1001/jama.2013.1937

[32] Serisier DJ, Martin ML, McGuckin MA (2013) Effect of long-term, low-dose erythromycin on pulmonary exacerbations among patients with non-cystic fibrosis bronchiectasis: the BLESS randomized controlled trial. JAMA 309: 1260–1267. 23532242 10.1001/jama.2013.2290

[33] Elborn JS, Tunney MM (2013) Macrolides and bronchiectasis: clinical benefit with a resistance price. JAMA 309: 1295–1296. 23532247 10.1001/jama.2013.2780

[34] Ogrendik M, Karagoz N (2011) Treatment of rheumatoid arthritis with roxithromycin: a randomized trial. Postgrad Med 123: 220–227. 21904105 10.3810/pgm.2011.09.2478

[35] Ogrendik M (2007) Effects of clarithromycin in patients with active rheumatoid arthritis. Curr Med Res Opin 23: 515–522. 17355733 10.1185/030079906X167642

[36] Wilson R, Wells AU (2012) Azithromycin in bronchiectasis: when should it be used? Lancet 380: 627–629. 22901872 10.1016/S0140-6736(12)61261-6

[37] Foster CS, Forstot SL, Wilson LA (1984) Mortality rate in rheumatoid arthritis patients developing necrotizing scleritis or peripheral ulcerative keratitis. Effects of systemic immunosuppression. Ophthalmology 91: 1253–1263. 6514289 10.1016/s0161-6420(84)34160-4

[38] Erhardt CC, Mumford PA, Venables PJ, Maini RN (1989) Factors predicting a poor life prognosis in rheumatoid arthritis: an eight year prospective study. Ann Rheum Dis 48: 7–13. 2923507 10.1136/ard.48.1.7 PMC1003667

[39] Turesson C, Jacobsson L, Bergström U (1999) Extra-articular rheumatoid arthritis: prevalence and mortality. Rheumatology (Oxford) 38: 668–674. 10461483 10.1093/rheumatology/38.7.668

[40] Gabriel SE, Crowson CS, Kremers HM, Doran MF, Turesson C, et al (2003) Survival in rheumatoid arthritis: a population-based analysis of trends over 40 years. Arthritis Rheum 48: 54–58. 12528103 10.1002/art.10705

[41] Turesson C, McClelland RL, Christianson TJ, Matteson EL (2007) Severe extra-articular disease manifestations are associated with an increased risk of first ever cardiovascular events in patients with rheumatoid arthritis. Ann Rheum Dis 66: 70–75. 16877533 10.1136/ard.2006.052506 PMC1798415

