# Supplementary figures and images for: Poor Survival in Rheumatoid Arthritis Associated with Bronchiectasis: A Family-Based Cohort Study
Source: PLoS One. 2014 Oct 13;9(10):e110066. doi: 10.1371/journal.pone.0110066 (PMC4195708; doi:10.1371/journal.pone.0110066)

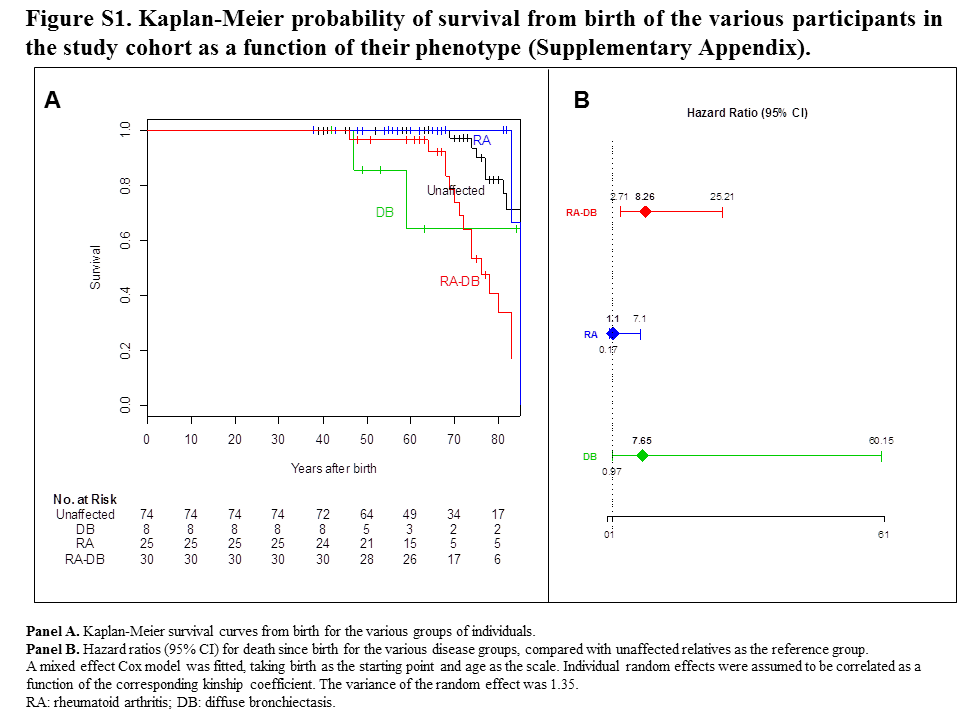

Supplement: Figure S1 — Kaplan-Meier probability of survival from birth of the various participants in the study cohort as a function of their phenotype. Panel A. Kaplan-Meier survival curves from birth for the various groups of individuals. Panel B. Hazard ratios (95% CI) for death since birth for the various disease groups, compared with unaffected relatives as the reference group. A mixed effect Cox model was fitted, taking birth as the starting point and age as the scale. Individual random effects were assumed to be correlated as a function of the corresponding kinship coefficient. The variance of the random effect was 1.35. RA: rheumatoid arthritis; DB: diffuse bronchiectasis. (TIF) [file pone.0110066.s001.tif]
